# The Nerve Element in Whooping Cough

**Published:** 1882-07

**Authors:** 


### THE NERVE ELEMENT IN WHOOPING COUGH.

The *Lancet* says : Of late years the profession has bestowed very little, if any, serious scientific attention on some of the commonest of common maladies. Whooping-cough is conspicuously among the neglected ills to which, notwithstanding the forgetfulness of the multitude of earnest clinical investigators, flesh is still heir. Many years ago the nerve-element in this troublesome and too often evil-working, if not in itself dangerous affection, engaged much consideration, and treatment was specially directed to its relief. It would be well if the investigation of this feature of the etiology of the affection could be resumed. The fact that pertussis belongs to the class of maladies which are communicable and "catching" does not take it out of the range of probability that the specific action of a morbific poison on the nerve-centres may be the efficient cause of the disease. Although the occurrence of the affection happening rarely more than once in the life of any individual may seem to point more directly to the fertilizing of latent germs in the organism than to any special excitation of the nerve-centres, we do not, as yet, know enough of the *modus operandi* of morbific influences—"germs" or poisons as we may call them—in the blood and the tissues to define the part which the nerve-centres play in the production of morbid phenomena. In any case, such relief is frequently obtained, even in the earliest stages of whooping-cough, from mild periodic counter-irritation over the whole length of the spinal column by a mustard poultice, which merely reddens the skin without vesication, that it would be well worth while to study this method closely from the therapeutic as well as the clinical standpoint. It certainly does good ; but how ? In cases where the mustard poultice, applied for six or eight minutes—not longer—

over the whole length of the spine immediately before putting the child to bed, every night for a week, or, in seriously spasmodic cases, a fortnight, does not procure a permanent amelioration of the cough, the effect of this remedy is enhanced by sponging the spine with iced water quickly, each successive morning. In cases where the paroxysms of cough seem to be repeated and to continue from sheer exhaustion of the nerve-centers, coffee, administered as a drink, will often stimulate the energy of the centres so as to put an end to the malady. These are practical points which require theoretical explanation.

